# Under-cover stabilization and reactivity of a dense carbon monoxide layer on Pt(111)†
†Electronic supplementary information (ESI) available: Additional XPS spectra and LEED images. See DOI: 10.1039/c8sc04461a


**DOI:** 10.1039/c8sc04461a

**Published:** 2018-12-03

**Authors:** Igor Píš, Elena Magnano, Silvia Nappini, Federica Bondino

**Affiliations:** a Elettra - Sincrotrone Trieste S.C.p.A. , 34149 Basovizza , Trieste , Italy . Email: igor.pis@elettra.eu; b IOM-CNR , Laboratorio TASC , 34149 Basovizza , Trieste , Italy . Email: bondino@iom.cnr.it; c Department of Physics , University of Johannesburg , Auckland Park 2006 , South Africa

## Abstract

The space between a metal surface and a two-dimensional cover can be regarded as a nanoreactor, where confined molecule adsorption and surface reactions may occur. In this work, we report CO intercalation and reactivity between a graphene-hexagonal boron nitride (h-BNG) heterostructure and Pt(111). By employing high resolution X-ray photoemission spectroscopy (XPS) we demonstrate the molecular intercalation of the full h-BNG overlayer and stabilization of a dense  *R*23.4°–13CO layer on Pt(111) under ultra-high vacuum at room temperature. We provide experimental evidence of a weakened CO–metal bond due to the confinement effects of the 2D cover. Temperature-programmed XPS results reveal that CO desorption is kinetically delayed and occurs at a higher temperature than on bare Pt(111). Moreover, CO partially reacts with the h-BNG layer to form boron-oxide species, which affect repeated CO intercalation. Finally, we found that the properties of the system towards interaction with CO can be considerably recovered using high temperature treatment.

## Abstract 2

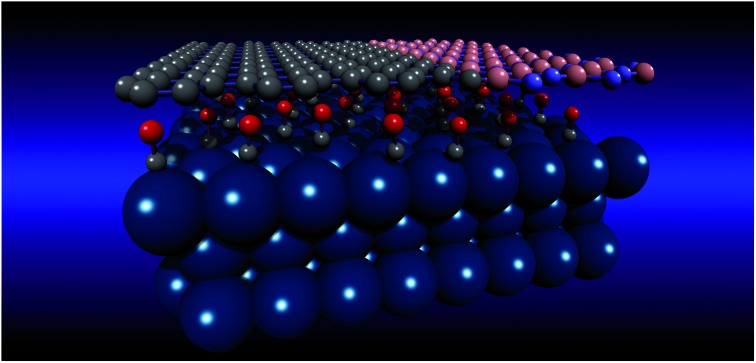
A dense CO overlayer on a Pt(111) surface under a 2D hybrid h-BN–graphene cover was studied.

## Introduction

A two-dimensional (2D) layer containing carbon (C), boron (B) and nitrogen (N) in a continuous honeycomb network is a promising new material with wide range applications in the fields of electronics, gas sensors, or heterogeneous catalysis. In the present work, using CO as a probing molecule, we investigate the suitability of the interface between a hybrid graphene (Gr) and hexagonal boron nitride (h-BN) 2D layer and a platinum substrate for chemical reactions confined under 2D covers.^1^–^4^ Confined chemistry under 2D materials may take advantage of the catalytic properties of the substrate, the geometric constraints, and the confinement field. This approach can stabilize active sites or modulate surface reactions, resulting in a fundamental effect on the catalytic performance. In addition, some 2D materials have exhibited catalytic promoting properties when used as catalyst supports.^5^,^6^ Thus, chemistry that occurs under a cover is an area of active research, as it could lead to unique applications in electronics, batteries, and other technologies.

It has been reported that 2D nanosheets, such as h-BN, graphene and graphitic g-C_3_N_4_ carbon nitride, when placed over a Pt(111) surface weaken the CO–metal bond and thus promote CO oxidation.^7^–^9^ However, little research has been performed on molecule intercalations and reactions under hybrid in-plane heterostructures, such as h-BNG. The presence of sp^2^ B–C and N–C bonds and specific h-BN–Gr domain boundaries could modulate interface reactions, leading to new chemical reaction pathways with respect to the pure graphene or pure h-BN counterparts.^10^ For instance, a h-BN–Gr one-dimensional interface has been found to be catalytically active in oxygen reduction reactions.^11^,^12^

Recently, we have demonstrated that it is possible to grow a graphenic atomically thin layer from a single precursor molecule using a bottom-up on-surface synthesis approach. By exposing a clean Pt(111) surface to dimethylamine borane (DMAB: (CH_3_)_2_NH·BH_3_), it is possible to grow either a C–B–N ternary layer with strongly hybridized B–N, B–C, C–C and C–N bonds or, alternatively, an ordered hexagonal boron nitride–graphene (h-BNG) single layer with complementary graphene and boron nitride domains.^13^,^14^ The h-BNG layer has proven to be a robust single-layer membrane with strong in-plane bonding and a weak interaction with the Pt substrate underneath. In this work, we investigate CO intercalation and thermally-induced desorption through the full h-BNG layer on Pt(111), employing high-resolution synchrotron-radiation soft X-ray photoelectron spectroscopy (XPS). The first part of the work is devoted to the CO intercalation underneath a full h-BNG layer grown on the Pt(111) substrate and in the second part of the work, we investigate the reaction between h-BNG, CO, and Pt during thermal annealing under ultra-high vacuum (UHV) conditions. The experimental results are compared with measurements performed on a clean Pt(111) surface and on a full graphene layer on Pt(111).

## Experimental

The experiments were carried out at the photoemission end-station of the Beamline for Advanced diCHroism (BACH) at the Elettra synchrotron facility in Trieste, Italy.^15^,^16^ The samples were prepared and characterized in an UHV chamber with a base pressure lower than 1 × 10^–9^ Torr. The Pt(111) single-crystal substrate (MaTecK, GmbH, 99.999% purity) was cleaned by 1.5 keV Ar^+^ ion sputtering, annealing in O_2_ (2 × 10^–7^ Torr) at 900 K for a few minutes, and vacuum annealing at 1000 K. The sample temperature was measured by an N-type thermocouple clamped to the edge of the Pt crystal. The cleanliness and structural quality of the surface were checked by photoemission spectroscopy and low energy electron diffraction (LEED).

To grow the hybrid layer of graphene and h-BN, the Pt(111) substrate was exposed to 150 L (1 L = 1 × 10^–6^ Torr for 1 s) of dimethylamine borane at a pressure of 5 × 10^–7^ Torr, while keeping the substrate temperature at 1000 K. This procedure leads to the formation of a continuous single layer composed of graphene and h-BN domains with a low percentage of impurities (<3%), such as boron carbides and some N and B single atoms incorporated into the graphene lattice. Lateral sizes of the domains are typically as large as a few hundred nm. More details on the growth, structure and chemical composition are described elsewhere.^13^,^14^,^17^ The h-BNG layers prepared for the present work comprised 62 ± 5 and 35 ± 5 at% graphene and h-BN phases, respectively. Single-layer graphene was grown by dosing 150 L of ethylene on Pt(111) kept at 1000 K.^18^

Adsorption of CO in the UHV chamber was performed at a partial pressure of 0.5–2 × 10^–7^ Torr. Exposure to high-pressure CO was carried out at room temperature (RT) in the load-lock of the experimental system with a base pressure of 5 × 10^–8^ Torr. After each high-pressure treatment, the sample was immediately transferred to the UHV chamber for further analysis. A low CO desorption rate of ∼0.04 ML per hour, calculated from XPS spectra intensities measured as a function of time, means that carbon monoxide desorption during the transfer (∼10 min) can be neglected. To further reduce CO desorption during the measurements, the photoemission spectra were acquired at a temperature below 250 K, except for the temperature-programmed experiments. Throughout this work, the amount of adsorbed or intercalated CO will be given with respect to the number of surface Pt atoms. One monolayer (ML) of Pt(111) corresponds to 1.5 × 10^15^ atoms per cm^2^. For comparison, the areal densities of atoms in graphene and h-BN are 3.82 × 10^15^ and 3.70 × 10^15^ atoms per cm^2^, respectively.

High-resolution synchrotron-radiation-excited XPS spectra were acquired using a VG-Scienta R3000 hemispherical analyser at an angle of 60° with respect to the X-ray incidence direction.^19^ The Pt 4f, C 1s, N 1s, and B 1s core levels were measured at a photon energy of 528 eV with a total energy resolution of 0.15 eV. A photon energy of 652 eV and a resolution of 0.22 eV were employed for the O 1s spectrum. The binding energies (BE) were calibrated with respect to the substrate Fermi edge and the error in their absolute values is estimated to be less than 0.1 eV. The core level spectra were recorded in normal emission geometry, except for Pt 4f, which was measured at an emission angle of 60° to enhance the relative intensity of the surface component. The high-resolution valence band spectra were recorded at a photon energy of 50 eV using linearly-polarized light with a polarization vector 30° off the surface plane. The C 1s and Pt 4f core levels were decomposed into their spectral components using Doniach–Šunjić asymmetric line shapes convoluted with a Gaussian function, and the other spectra were fitted with Voigt line shapes. A Shirley-type background was subtracted. Errors in the relative positions of the spectral components were estimated from the peak fitting procedures.

## Results and discussion

### CO intercalation under a full h-BNG layer

First, the h-BNG/Pt(111) sample was exposed to CO in the UHV chamber. To monitor CO intercalation between h-BNG and Pt(111) or the potential adsorption of CO on top of h-BNG, high-resolution Pt 4f, C 1s, O 1s, N 1s, and B 1s spectra were measured before and after each CO exposure step. The O 1s and C 1s regions were selected to monitor the presence of adsorbed CO, whereas the Pt 4f region was chosen to observe CO intercalation, as the molecule chemisorption induces a significant core-level shift of the Pt 4f surface component. The h-BNG/Pt(111) was exposed to CO at various temperatures. No signs of adsorption or intercalation were detected in the investigated temperature range 160–300 K at a CO pressure of 2 × 10^–7^ Torr and up to 400 L exposure. The sample was then moved to the load-lock, which was backfilled with CO up to a pressure of 100 Torr. The high-pressure exposure was done at RT and the sample was transferred to the analysis UHV chamber and cooled down below to 250 K shortly thereafter. The first signs of intercalation were observed at pressures higher than 10 Torr. Exposure to 50 Torr for 10 min was found to be sufficient to achieve CO saturation intercalation under the h-BNG layer. Saturation was confirmed by observing no further changes in the XPS spectra with a prolonged CO dose. For comparison, a full layer of graphene grown on the same Pt(111) substrate (see the ESI†) was exposed to CO under the same conditions, but only a small amount of intercalated CO was detected. One hour of exposure to 100 Torr was necessary to achieve CO saturation intercalation at the Gr/Pt(111) interface at RT. It has been proposed that the diffusion of molecular species into interfaces between metals covered by full sp^2^-hybridized layers happens through structural defects, such as pinholes,^20^ grain boundaries^21^,^22^ or wrinkles.^23^ Thus, the lower intercalation pressure for h-BNG is not surprising, as the heterostructure is expected to have more and different structural defects, such as h-BN–Gr boundaries, compared to the graphene layer. In fact, a slightly more defective h-BNG layer prepared at a lower temperature required lower CO saturation exposure (see Fig. S1 in the ESI†).

**Fig. 1:**
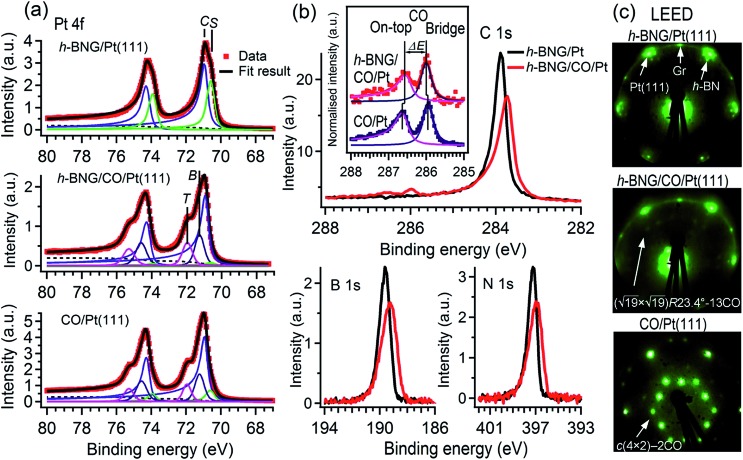
(a) Pt 4f XPS spectra deconvoluted into crystal bulk (*C*), surface (*S*) components and Pt surface species bonded to CO chemisorbed in on-top (*T*) and bridge (*B*) sites. (b) C 1s, B 1s, and N 1s spectra from the full h-BNG layer measured before and after high-pressure CO exposure at RT (*p*(CO) = 50 Torr, 10 min); the inset compares the C 1s peaks of CO adsorbed on bare Pt(111) and CO intercalated at the h-BNG/Pt(111) interface. (c) LEED diffraction patterns recorded with electron energy *E*_0_ = 78 eV.

Fig. 1a shows the Pt 4f spectrum from freshly prepared h-BNG/Pt(111) and from the same sample after high-pressure CO saturation exposure at RT. A spectrum from the bare Pt(111) surface exposed to a CO saturation dose (60 L at RT and *p*_CO_ = 2 × 10^–7^ Torr) is also included for comparison. Two distinct components are clearly visible in the spectrum from the h-BNG covered Pt(111) surface. The component *C* at a BE of 70.9 eV corresponds to Pt crystal bulk atoms and the second component *S* shifted by Δ*E*_BE_ = –0.36 ± 0.02 eV, is assigned to the topmost Pt(111) atoms. Owing to the weak interaction with the above lying h-BNG layer, the intensity and shift of this component is comparable with the surface component of the bare Pt(111) surface (ESI, Fig. S2a†). On the other hand, the *S* component is significantly reduced after the high-pressure exposure to CO. Meanwhile, a high-energy shoulder appeared in the Pt 4f spectrum. The overall shape is similar to the one from CO covered Pt(111) and the shoulders after the CO chemisorption can be well fitted with new components at BE = 72.0 and 71.3 eV, which have been previously attributed to Pt atoms bonding to CO molecules sitting in on-top and bridge sites, respectively.^24^ The relative intensities of the *S*, *T* and *B* components can be used to estimate the CO coverage. The calculated coverage *θ*_CO_ = 0.53 ± 0.02 ML on bare Pt(111) is in good agreement with the expected value reported previously.^25^ The Pt surface component on h-BNG/CO/Pt(111) is more profoundly reduced, which corresponds to a higher coverage *θ*_CO_ = 0.67 ± 0.02 ML (see Fig. S2 in the ESI† for more details).

The CO intercalation is also accompanied by changes in the C 1s, N 1s, and B 1s spectra, as reported in Fig. 1b. The pristine h-BNG layer exhibits C 1s, N 1s, and B 1s main peaks at 283.9 eV, 397.2 eV, and 189.6 eV respectively, which are characteristic of sp^2^ hybridized graphene and h-BN on platinum.^13^ Core-level shifts appeared in all three spectra after the CO dose, which means that CO molecules were intercalated under both the graphene and h-BN parts of the hybrid layer. Negative core level shifts are evidence of a 2D epitaxial overlayer/metal interface subjected to molecule intercalation, as the intercalated molecules reduce the hybridization between the epitaxial overlayer and the metal substrate.^1^,^26^ The integrated peak areas remain constant within the limits of error, but they become broader, which can be modeled by a new component with a core-level shift Δ*E*_BE_ = –0.18 ± 0.03 eV in the case of C 1s, and Δ*E*_BE_ = –0.54 ± 0.06 eV in the case of both the N 1s and B 1s spectra. These values are in good agreement with the core level shifts previously reported for CO intercalated under pure graphene or h-BN single layers, respectively.^7^,^8^

Two other weak, but well resolved, components appeared in the C 1s region at the high BE side, as shown in Fig. 1b. The higher BE peak is attributed to CO molecules adsorbed on Pt(111) superficial atoms at on-top sites and the lower BE peak is due to CO at bridge sites.^24^,^27^–^29^ Corresponding O 1s peaks appeared at 532.6 and 530.9 eV, as reported further. The CO C 1s components are displayed in more detail in the inset of Fig. 1b, where the contribution from graphene has been subtracted. The C 1s spectrum corresponding to pure Pt(111) exposed to a low-pressure CO saturation dose at RT is displayed for comparison. The two spectra are normalised to the peak heights. Small but discernible core level shifts for CO under the h-BNG compared to CO on bare Pt(111) are observed. The CO-bridge peak shifts from 285.9 eV to 286.0 eV and its separation Δ*E* from the on-top peak decreases from Δ*E* = 0.68 ± 0.02 to Δ*E* = 0.57 ± 0.03 eV. These changes can be considered as another fingerprint of intercalated CO. Since the CO molecules adsorb on the surface *via* the C atoms, the C 1s core level shifts are sensitive to the local environment and they can be influenced by the lateral shifts in the CO positions from their regular high symmetry adsorption sites. The apparent decrease in the CO C 1s peak separation could be due to such lateral displacements and molecular tilt from the surface normal, as has been predicted for a dense CO overlayer on Pt(111).^24^

A high-density CO adsorption phase on Pt(111) has been reported under UHV at low temperatures,^30^ in high CO partial pressures at room temperature^24^,^31^,^32^ and on Pt electrodes in electrochemical cells.^33^ Although several densely packed CO structures have been observed, the 
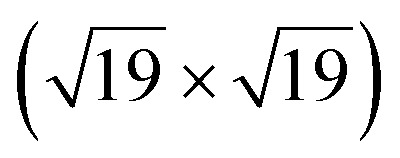
 *R*23.4°–13CO structure (hereafter 
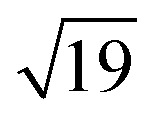
–13CO) consisting of 6 CO-bridge and 7 CO-top sites with a theoretical coverage of 0.68 ML has been the most frequently observed. Such a structure appeared also in our case, as indicated by the LEED pattern shown in Fig. 1c.^34^ The LEED pattern from freshly grown h-BNG/Pt(111) displays typical diffraction spots corresponding to Pt(111)–(1 × 1) surrounded by a Moirè superstructure from single-oriented epitaxial h-BN and arc-shaped diffraction centered at 30° from the Pt(1 × 1) spots originating from graphene.^13^ The Moirè pattern is considerably attenuated after the CO treatment due to the h-BN–substrate decoupling induced by CO.^26^ At the same time, spots of the 
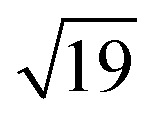
–13CO structure are observed. The LEED pattern from CO saturation coverage on Pt(111) at RT under UHV is also displayed. The pattern corresponds to a well-characterized *c*(4 × 2)–2CO structure with 0.5 ML coverage where equal amounts of molecules populate the two types of sites,^25^ which is consistent with the coverage determined from the Pt 4f XPS spectra.

It is worth noting that the excessive amount of CO quickly desorbs once Pt(111)^24^ or Pt(111) covered by submonolayer graphene and h-BN islands^7^ are exposed to high-pressure CO and subsequently placed under UHV at RT. In the case of h-BNG/CO/Pt(111), we observed a slow CO desorption rate at RT (0.04 ML per hour at CO saturation coverage) and no CO desorption for at least 12 hours at *T* ≈ 200 K. This under-cover stability of CO is in line with the relatively high intercalation pressure reported above. A similar stabilization of a dense CO adlayer has also been reported for monolayer Gr/Ru^35^ and h-BN/Pt(111) interfaces.^36^ In contrast to the submonolayer graphene or h-BN islands,^7^ no open edges are present on the full h-BNG layer and the CO intercalation and desorption happen on defects with apparently higher diffusion barriers.

**Fig. 2:**
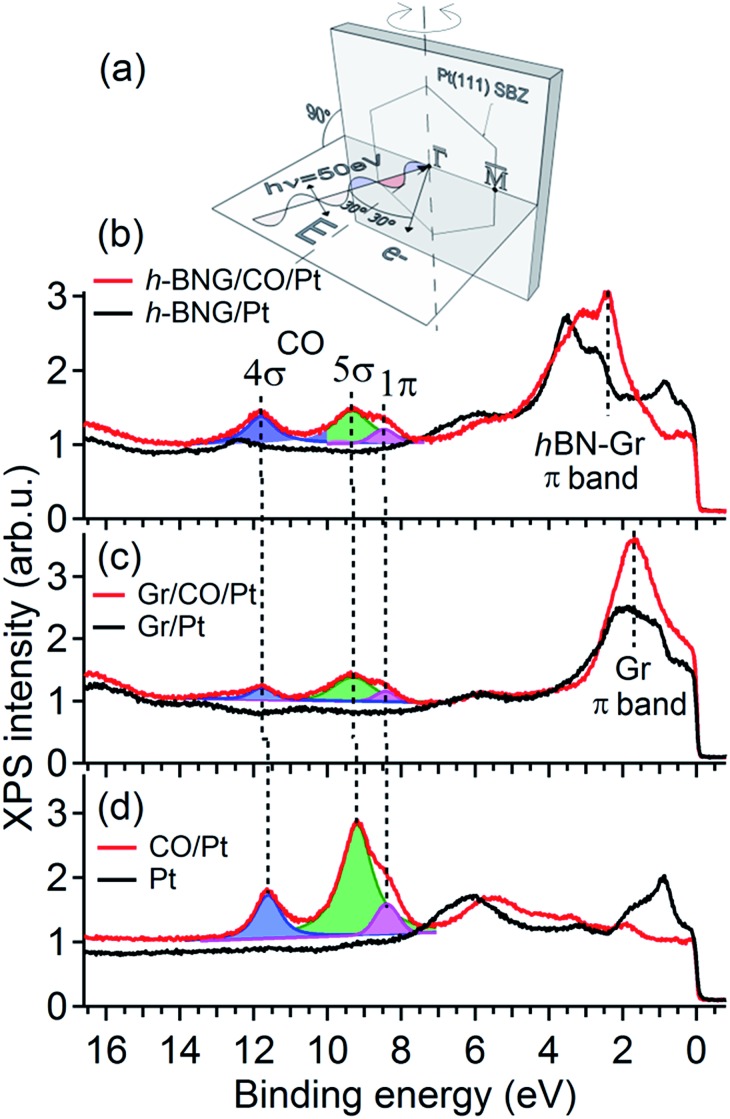
Valence band spectra acquired with photon energy *hν* = 50 eV before and after CO saturation doses at RT. (a) Schematic diagram of the experimental geometry. The substrate surface Brillouin zone (SBZ) represents the sample orientation. (b) Full h-BNG layer on Pt(111); *p*(CO) = 50 Torr, 10 min. (c) Full graphene layer on Pt(111); *p*(CO) = 100 Torr, 1 h. (d) Pt(111); *p*(CO) = 5 × 10^–8^ Torr, 20 min. The dashed lines are guides for the eye. Angle resolved valence band spectra are shown in the ESI.†

To further investigate the confinement effect of the h-BNG cover on the CO–Pt bond, high-resolution valence band spectra were recorded (Fig. 2). The photon energy, emission angle, and light polarization vector orientation were chosen to emphasize the emission from CO molecular orbitals and to avoid overlap with graphene and h-BN valence band features (Fig. 2a).

Fig. 2b shows the valence band spectra before and after CO intercalation under a freshly grown full h-BNG overlayer on Pt(111). The results are compared with those of CO intercalated under full single-layer graphene on Pt(111) (Fig. 2c) and CO adsorbed on bare Pt(111) (Fig. 2d). Room temperature CO saturation was reached in each case. After the gas exposure, three well-known CO derived peaks corresponding to the bonding 5σ and non-bonding 4σ, 1π molecular orbitals of CO on Pt(111) appeared in the spectra of all of the samples.^37^–^39^ According to previous studies,^40^–^43^ the separation Δ(4σ–5σ) is proportional to the CO–metal bond length, and increases significantly with decreasing CO adsorption energy. Moreover, the energy difference Δ(4σ–1π) has been found to be proportional to the carbon–oxygen bond distance and an increase in this difference typically corresponds to a lower CO dissociation barrier.^40^,^44^ The measured 4σ, 5σ, and 1π peak positions and their separations for the intercalated CO are summarized in Table 1.

**Table 1:** Binding energies (in eV) of CO molecular orbitals and their separation Δ. The errors in the absolute values of the BE, deduced from the fitting procedure, are estimated to be less than 0.05 eV

Sample	Molecular orbital				
	4σ	5σ	1π	Δ(4σ–5σ)	Δ(4σ–1π)
h-BNG/CO/Pt(111)	11.80	9.35	8.47	2.45	3.33
Gr/CO/Pt(111)	11.77	9.30	8.42	2.47	3.35
CO/Pt(111)	11.62	9.21	8.39	2.41	3.23

The CO confined between Pt and the h-BNG cover show a subtle increase in both Δ(4σ–5σ) and Δ(4σ–1π) compared to pure Pt(111), which are almost equal to CO intercalated between Pt and the single-layer graphene. Although the changes are close to the experimental error they indicate a slight weakening of the C–O bonds and the CO–metal interactions. The small variations in the bond strength are in good agreement with recent studies of CO adsorption, desorption and oxidation on Pt(111) covered by graphene and h-BN sheets.^7^,^8^ Yao and coworkers^7^ demonstrated that the confinement effect of the graphene overlayer lowers the apparent activation energy of the CO oxidation. The DFT calculations performed on graphene covered Pt(111) show that the electronic interaction between the graphene and CO molecule lowers the CO–Pt adsorption energy and, at the same time, leads to electron loss in the C–O bond. Our valence band study indicates that the hybrid h-BNG layer influences the CO chemisorption in a similar way.

### CO desorption from the h-BNG/Pt(111) interface

**Fig. 3:**
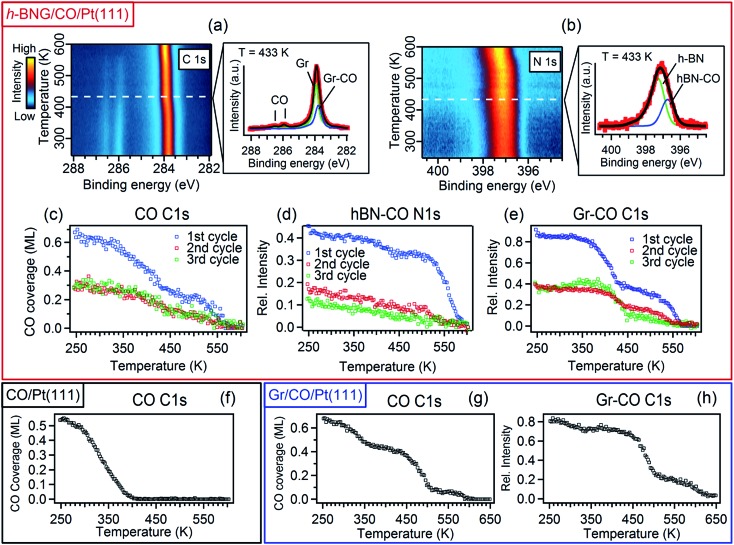
Image plots of C 1s (a) and N 1s (b) temperature-programmed XPS spectra (heating rate 3 K min^–1^) for a full layer of h-BNG on Pt(111) after high-pressure CO (50 Torr) saturation intercalation at RT and representative line profiles extracted at the indicated temperature. In the fitting, the green coloured components correspond to pristine graphene and h-BN on Pt, blue coloured components, labeled as Gr–CO and hBN–CO, represent the CO intercalated graphene and h-BN fractions, respectively. (c) CO coverage thermal evolution derived from the areas of the respective C 1s peak components. (d and e) Thermal evolution of the hBN–CO N 1s and Gr–CO C 1s components determined from the spectra in (a). The intensities are normalised to the graphene and h-BN peak areas before the first intercalation. (f) TP-XPS for the CO saturation layer adsorbed on bare Pt(111) at RT. (g and h) TP-XPS for a full single layer graphene on Pt(111) after high-pressure CO intercalation at RT. The CO coverage (g) and relative intensity of the Gr–CO C1s component (h) are determined from C 1s spectra in the same manner as for h-BNG. All XPS spectra were acquired with photon energy *hν* = 528 eV.

To investigate the interaction between h-BNG/Pt(111) and intercalated CO at elevated temperatures, the sample was annealed under UHV up to 610 K. When cooled down to RT, the high-pressure intercalation and annealing was repeated. In total, 3–4 cycles were performed on two freshly grown samples. Figure 3 shows the results of temperature-programmed XPS (TP-XPS) measurements obtained with a linear heating rate of 3 K min^–1^. Peak decomposition of the C 1s and N 1s spectra (Fig. 3a and b) allowed the separate characterization of the desorption of the CO molecules intercalated at the Gr/Pt and h-BN/Pt interfaces. The CO coverage, determined from the total area of the CO C 1s components, is plotted against the heating temperature in Fig. 3c (both CO components exhibited similar thermal evolution, as shown in Fig. S5 of the ESI†). The CO signal decrease with the temperature is mostly due to CO desorption, but partially also because of a reaction with the h-BNG cover, as will be shown below. The desorption kinetics is very different from that of the CO/Pt(111) reference sample shown in Fig. 3f. It is worth noting that a large amount of the confined CO remains chemisorbed on Pt(111) at significantly higher temperatures compared to the bare Pt(111). We showed above that the CO adsorption energy under the cover is similar or lower than on Pt(111). Thus, other kinetic parameters must be responsible for the higher desorption temperature. In fact, a lower CO desorption temperature is observed from the Pt(111) surface covered by a submonolayer of h-BNG, as reported in the ESI, Fig. S5(d and e).† A similar decrease in the desorption temperature has also been observed on a submonolayer of h-BN/Pt(111)^8^ and Gr/Pt(111).^7^ It was demonstrated that the main channels for CO diffusion under both h-BN and graphene covers are opened island edges. Such channels are absent in the full layer coverage. Therefore, similarly as for the higher intercalation pressures, we ascribe the higher desorption temperatures from underneath the full layer h-BNG to the structural quality, which is related to the amount and nature of the structural and topological defects. To further support this hypothesis, we also performed TP-XPS measurements on Pt(111) covered by h-BNG grown at a slightly lower temperature (∼950 K), which exhibited broader photoemission core level spectra, indicating a more defective layer accompanied by increased hybridization with the platinum substrate.^14^,^45^ Indeed, a lower desorption temperature was measured (Fig. S1 in the ESI†).

Furthermore, we propose that the structural defects could be at the origin of different CO desorption temperatures under the h-BN and graphene phases in the h-BNG heterostructure, as shown in the thermal evolution of the Gr–CO and hBN–CO C 1s and N 1s components in Fig. 3d and e. While the main CO desorption from Pt under h-BN occurs between 550 and 590 K, desorption from Pt under graphene begins at a temperature of 350 K. Previous microscopic investigations^13^,^17^ showed that h-BN domains grow almost exclusively in one azimuthal orientation with respect to the substrate lattice, whereas smaller graphene domains with different azimuthal orientations are formed. The same phase composition of the studied samples was confirmed by LEED. The LEED pattern shown in Fig. 1c shows an arch-shaped diffraction spot from the graphene rotational domains and we ascribe the lower CO desorption to these domains or their boundaries. For comparison, the same TP-XPS measurements were performed on single-layer graphene on Pt(111) after CO saturation intercalation and the results are plotted in Fig. 3g and h. The difference in the desorption kinetics compared to those of h-BNG can be related to different rotational domains dominated by the R19° phase (Fig. S4 in the ESI†)^18^,^46^ and to the absence of h-BN–Gr boundaries. Another effect contributing to the higher CO desorption temperature from under h-BN than from under graphene is the thermally-induced oxidation of boron, as discussed below.

**Fig. 4:**
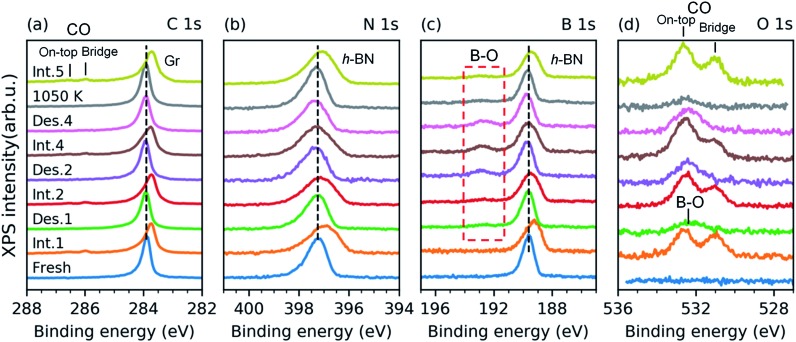
XPS C 1s (a), N 1s (b), B 1s (c), and O 1s (d) spectra from a freshly grown full h-BNG layer on Pt(111) (bottom) followed by repeated cycles of room temperature CO intercalation (Int.) at *p*(CO) = 50 Torr for 10 min and thermal desorption (Des.) at *T* = 610 K. The sample was annealed at *T* = 1050 K for ∼5 min after the fourth cycle.

We have investigated the reversibility by alternating the CO intercalation at RT and temperature-programmed annealing up to 610 K. The difference between the first and second TP-XPS spectrum (Fig. 3c–e) indicates irreversible changes. The total CO intercalation capacity drops to half and even more under the h-BN phase. Explanation of this irreversible change can be found in the B 1s and O 1s core-level spectra recorded after each intercalation and desorption step, as reported in Fig. 4.

The spectra presented in Fig. 4 were obtained from a freshly grown sample that exhibited spectral features and a composition equal to that of the first sample. After the first annealing step at 610 K under UHV, no CO spectral features are present in the C 1s or the O 1s spectra. However, new components appear in the O 1s and B 1s spectra (Fig. 4c and d). Both peaks further grow upon an increase in the number of intercalation-annealing cycles. This correlation, as well as the BE values of 532.4 and 192.7 eV for O 1s and B 1s, respectively, unambiguously imply the formation of B–O bonds upon reaction with CO.^17^,^47^–^50^ No oxidation of h-BN by CO has been reported on pure h-BN/Pt(111), thus we infer that the reaction has taken place at the ternary interface between h-BN, graphene, and Pt. XPS spectra of B 1s, N 1s, and C 1s exhibit peak broadening after the CO desorption, especially at the higher binding energy sides (Fig. 4a–c), which indicates a deterioration in the quality of the h-BNG layer accompanied by increased hybridization with the metal substrate.^45^ Moreover, the C 1s peak also exhibits a broadening on the lower energy side, at a BE characteristic of graphene vacancies,^51^,^52^ as well as atomic carbon on Pt (Fig. S6 in the ESI†), which could be a byproduct of the reaction between CO and the h-BNG layer.

**Fig. 5:**
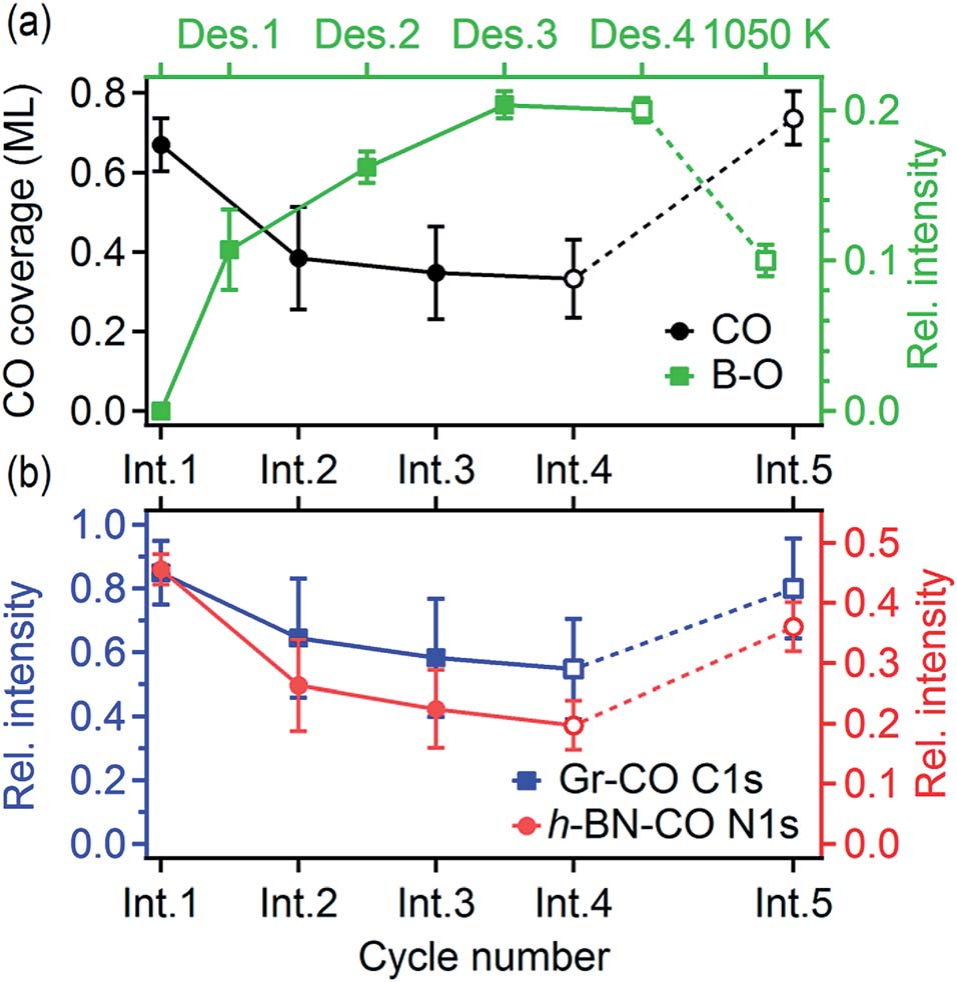
(a) CO coverage and the amount of boron oxides; (b) relative amount of CO intercalated graphene and h-BN displayed as a function of the intercalation/desorption cycle number. The relative intensities, determined from the XPS spectra fitting, are normalised to the Gr and h-BN peak areas before the first intercalation. The last desorption step at *T* = 610 K was followed by annealing at *T* = 1050 K. The lines serve as guides for the eye.

The rate of B–O growth and the rate of CO coverage decay slowed down upon an increase in the number of cycles, as shown in Fig. 5. If the active centers are h-BN–Gr boundaries, the progressive deactivation could be explained by the passivation of these particular line defects by oxygen. Other active centers could be point defects at the Pt surface,^53^ where CO dissociation takes place first, followed by atomic oxygen surface diffusion and reaction with the h-BN phase. The atomic carbon as the second product of the CO dissociation remains attached to the Pt reactive sites inhibiting further dissociation. The boron impurities (<2 at%, as determined from the XPS measurements), could also contribute to the dissociation, as they can substantially influence the adsorption properties of transition metal surfaces.^54^–^56^ Anyhow, the new oxygen-containing species and atomic carbon formed after the reaction apparently impede the CO diffusion into the h-BNG/Pt(111) interface, thus reducing the amount of chemisorbed CO. This effect is more pronounced in the case of h-BN (Fig. 5b), which could be ascribed to oxygen-terminated h-BN edges blocking CO intercalation.

We found that the ability of h-BNG/Pt(111) to store high CO coverage can be recovered by high temperature treatment. While no changes in the XPS spectra were detected after step-wise vacuum annealing at 800 K for 10 minutes, a profound reduction in the amount of boron oxide was observed after the annealing at *T* > 1000 K (Fig. 4c and d). The high-pressure CO intercalation after the temperature treatment again resulted in the formation of a high-coverage CO phase, as implied from the core-level peak shifts in Fig. 4 and CO XPS spectral component intensities reported in Fig. 5.

## Conclusions

We have investigated the intercalation and thermal desorption of CO from Pt(111) under a hybrid single layer of graphene and h-BN. High CO doses (∼10^10^ L at *p*_CO_ = 10–100 Torr) were found to achieve saturated CO intercalation of the full h-BNG cover at RT. A dense 
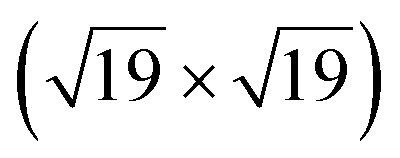
 *R*23.4°–13CO adlayer chemisorbed on Pt is formed under these conditions and it remains trapped at the interface under UHV conditions. High-resolution valence band photoemission spectroscopy provides experimental evidence of a slight weakening of the CO–Pt chemisorption bond confined under the h-BNG layer. Moreover the 2D cover profoundly alters the CO desorption kinetics and a large amount of CO is found to be chemisorbed on the Pt surface at temperatures much higher than those of bare Pt(111). The h-BNG overlayer acts as an effective barrier that confines CO in a 2D interface. Repeated cycles of CO intercalation and thermal desorption revealed irreversible changes in the h-BNG layer due to the thermally induced reaction with CO. Part of the intercalated CO molecules dissociates at ternary h-BN–Gr–Pt and the atomic oxygen reacts with boron. The reaction is hampered after a couple of cycles and the intercalation–desorption appears to be stabilized afterwards, although only with half the CO adsorption capacity. The system can be regenerated to a large extent by high-temperature treatment.

We showed that gas molecules such as CO can intercalate through the h-BNG 2D layer at room temperature and remain trapped underneath for several hours even under UHV conditions. Thus, mixed 2D layers, such as in-plane h-BN and graphene heterostructures, can also act as an effective barrier to confine gas molecules in a 2D interface. The h-BNG layer when also grown on other metals could serve as a membrane facilitating the investigation of mechanisms and fundamental steps in high-pressure and high-temperature catalytic processes. Furthermore, metallic atoms can be intercalated under h-BNG,^17^ which opens up the opportunity to further tune catalysis under two-dimensional materials.

## Conflicts of interest

There are no conflicts to declare.

## Supplementary Material

Supplementary information
