# Supplementary material for: Under-cover stabilization and reactivity of a dense carbon monoxide layer on Pt(111)
Source: Chem Sci. 2018 Dec 3;10(6):1857–65. doi: 10.1039/c8sc04461a (PMC6371755; doi:10.1039/c8sc04461a)
Supplement: Supplementary file 1 [file SC-010-C8SC04461A-s001.pdf]

Electronic Supplementary Material (ESI) for Chemical Science.
This journal is © The Royal Society of Chemistry 2018

Electronic Supplementary Information

Under-cover stabilization and reactivity of a dense carbon monoxide layer on Pt(111)

Igor Píš[a,b,*], Elena Magnano[b,c], Silvia Nappini[b], and Federica Bondino[b,*]

[a]*Elettra - Sincrotrone Trieste S.C.p.A., S.S. 14-km 163.5, 34149 Basovizza, Trieste, Italy*
[b]*IOM-CNR, Laboratorio TASC, S.S. 14-km 163.5, 34149 Basovizza, Trieste, Italy*
[c]*Department of Physics, University of Johannesburg, PO Box 524, Auckland Park 2006, South Africa*

Full *h*-BNG layer on Pt(111) prepared at T = 950 K

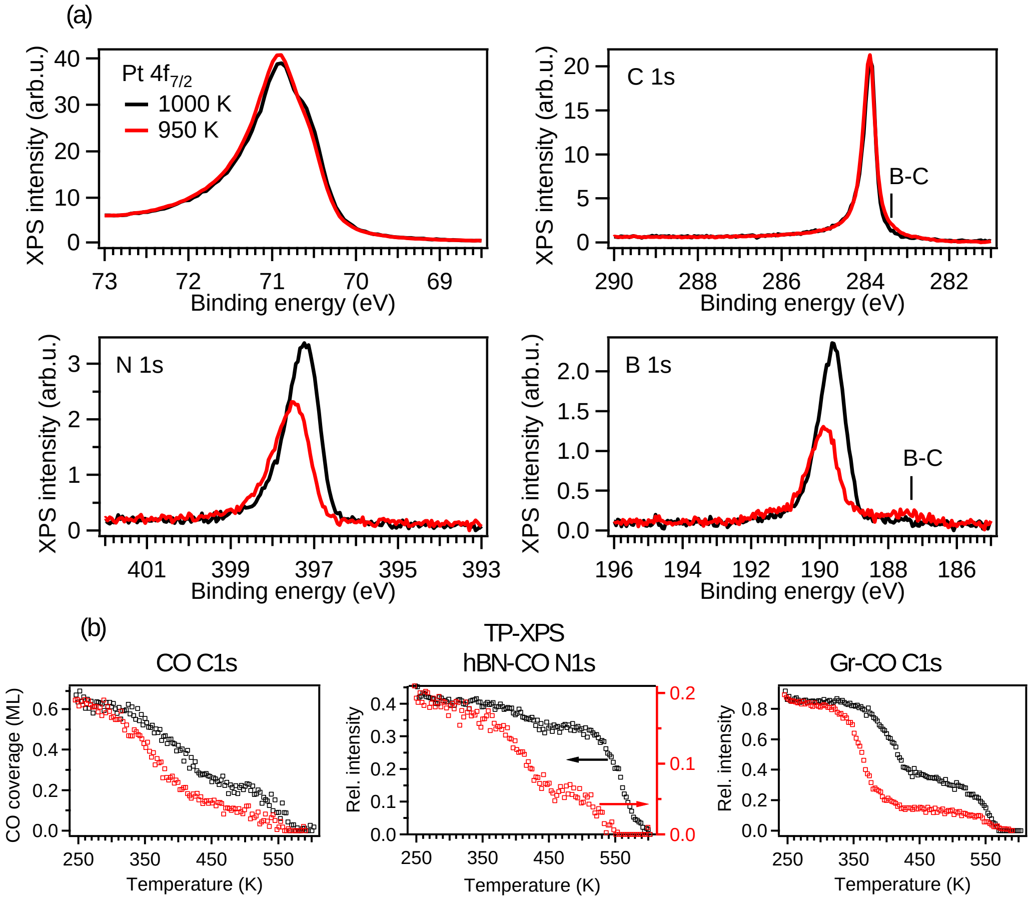

Fig. S1. (a) XPS Pt 4f, C 1s, N 1s, and B 1s spectra for full *h*-BNG layer prepared on Pt(111) substrate at two different temperatures, 1000 K (black curves) and ~950 K (red curves). B1s and N 1s spectra of the latter one are shifted to higher BE due to an increased hybridization with the Pt substrate, which also caused the lower relative intensity of the weakly interacting Pt $4f_{7/2}$ component at the low binding energy side. Signs of non-stoichiometric boron carbide are present both in the B 1s and in the C 1s spectra. (b) Thermal evolution of the CO coverage, hBN-CO N 1s and Gr-CO C 1s component intensities determined from the temperature-programmed XPS performed after room temperature CO saturation intercalation. All spectra were acquired with photon energy hν = 528 eV.

Table S1. Comparison between *h*-BNG grown on Pt(111) at T=1000 K and 950 K, respectively.

**Sample**	***h*-BNG/Pt(111) @ 1000 K**	***h*-BNG/Pt(111) @ 950 K**
**Composition**	62% Gr, 35% *h*-BN, 1.5% B_xC_y	69% Gr, 28% *h*-BN, 3.4% B_xC_y
**CO saturation dose**	50 Torr, 10 min	10 Torr, 10 min
**CO saturation coverage**	0.67 ML	0.64 ML

CO coverage determination

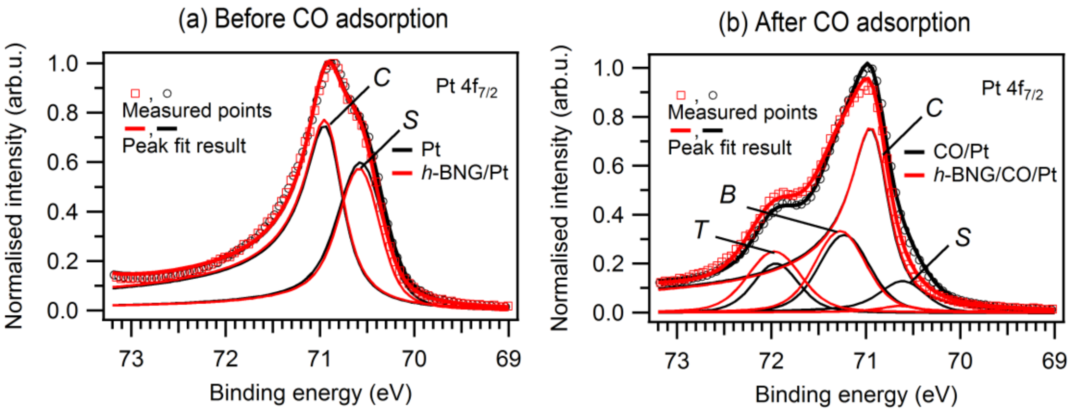

Fig. S2. The comparison between the Pt $4f_{7/2}$ XPS spectra of Pt(111) (red curves) and the *h*-BNG covered Pt(111) (black curves) surface before and after CO saturation doses at room temperature. The spectra are normalised to their integrated intensities. (a) The spectra before the CO exposure exhibit almost identical relative intensity ratio between the bulk (*C*) and surface (*S*) component. (b) The spectra after CO chemisorption. The intensity of the surface component *S* is reduced due to the chemical shift towards higher energies upon interaction with CO molecules. Component *T* corresponds to Pt atoms with CO molecules sitting on-top of them, *B* component is assigned to Pt atoms bonded to CO in bridge sites. Considering that two Pt surface atoms are affected by one CO molecule in the bridge site and one Pt surface atom by one CO molecule in on-top site, the CO coverage Θ can be determined from *S*, *T*, *B* peak areas: $\Theta = (B/2+T)/(B+T+S)$. This formula yields the coverage Θ_{CO} = 0.53±0.02 ML for the Pt(111) surface and Θ_{CO} = 0.67±0.02 ML for *h*-BNG covered Pt(111), where the error is deduced from the fitting procedure. All spectra were measured at the emission angle of 60° and a photon energy of 528 eV.

Angle-resolved photoemission of the valence band

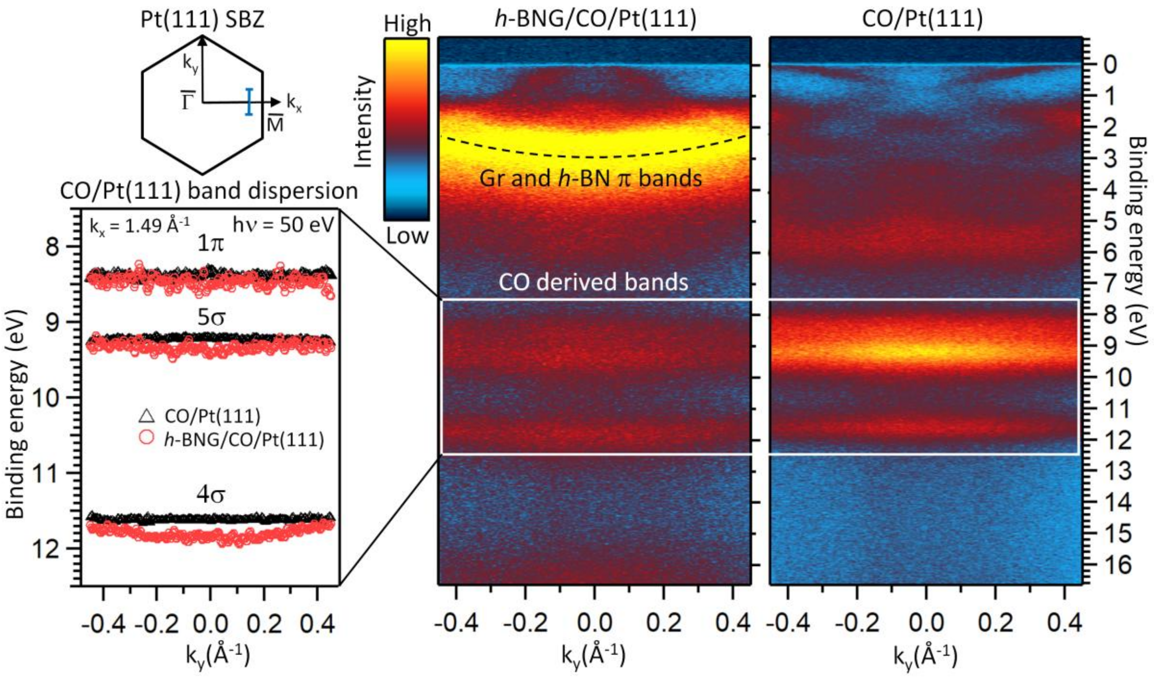

Fig. S3. Band dispersion of the CO derived valence band states along the direction indicated (blue line) in the Pt(111) surface Brillouin zone (SBZ). The angle resolved photoemission valence band maps were taken after room temperature CO saturation doses. Note the increased 4σ, 5σ band widths for *h*-BNG/CO/Pt(111). Although the influence of the *h*-BNG layer is not excluded here, the bandwidth is generally proportional to stronger CO–CO lateral interactions in densely packed CO structures.[1]

Single-layer graphene on Pt(111)

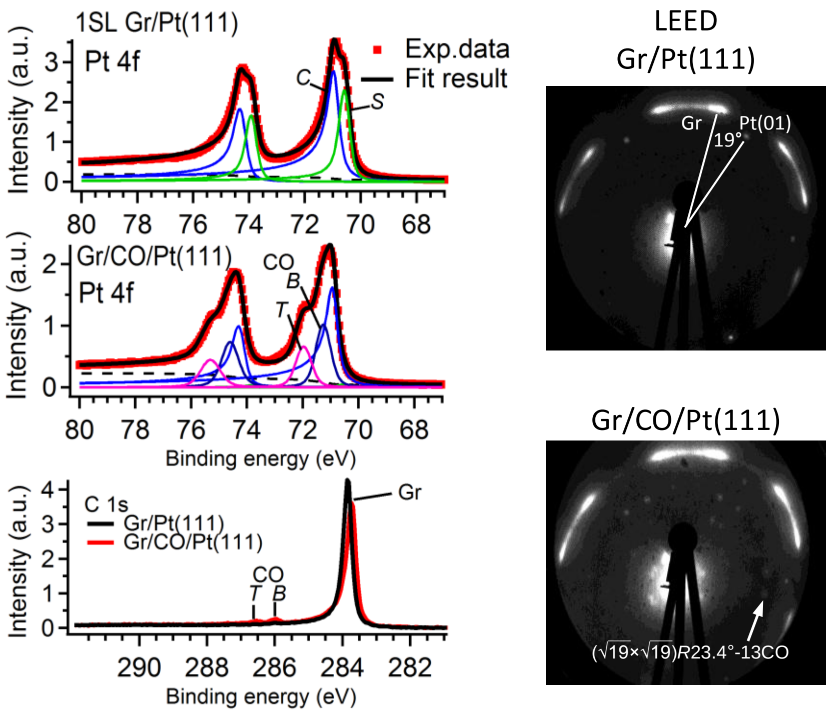

Fig. S4. XPS spectra and LEED patterns obtained from a full single-layer (SL) graphene on Pt(111) before and after high-pressure CO dose. Graphene was grown by dosing 150 L of ethylene on Pt(111) kept at 1000 K. CO dose was performed at room temperature at p(CO) = 100 Torr for 1 hour. XPS spectra were acquired with photon energy hν = 528 eV, LEED patterns with electron energy E_0 = 78 eV.

Site-dependent CO desorption determined from temperature-programmed XPS

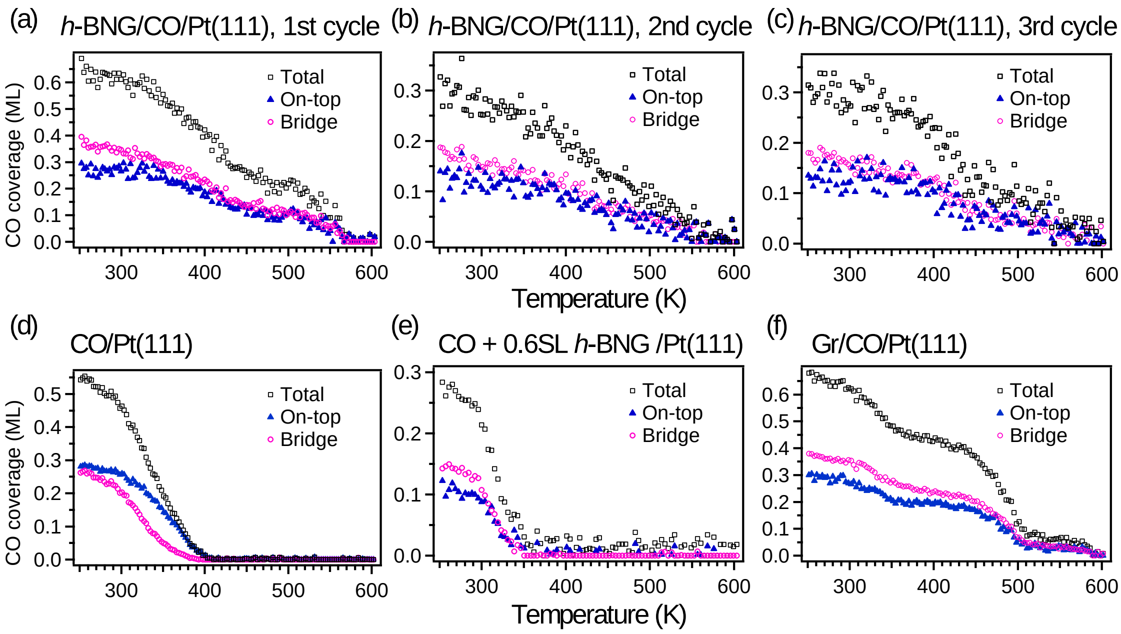

Fig. S5. Thermal evolution of CO coverage determined from high-resolution temperature programmed XPS C 1s spectra (heating rate 3 K/min), which where decomposed into components corresponding to CO chemisorbed on Pt(111) in on-top and bridge sites.(a–c) CO desorption from Pt(111) covered by full *h*-BNG layer exposed to high-pressure CO (p_{CO} = 50 Torr, 10 min). (d) Desorption of c(4×2) – 2CO overlayer prepared on bare Pt(111) by dosing 40 L of CO at p(CO) = 5×10^{-8} Torr. Pt(111) was kept at room temperature during the dose. Then it was cooled down to 250 K. (e) Desorption of CO from Pt(111) partially covered with *h*-BNG submonolayer (0.65 of the single layer - SL). The sample was exposed to 40 L of CO at T = 156 K and p(CO) = 1×10^{-7} Torr. Note the decrease of the desorption temperature by about 25 K in comparison with that of bare Pt(111). (f) CO desorption from Pt(111) covered by full single-layer graphene after the high-pressure CO treatment (100 Torr for 1h).

The faster desorption of CO from bridge sites in the case of CO/Pt(111) is in agreement with lower adsorption energy in comparison with on-top sites, as reported previously.[2] On the contrary, samples with *h*-BNG exhibit similar desorption kinetics for both CO in bridge and on-top sites, which indicates that the 2D cover is reducing the difference between adsorption energies of CO in the two sites.

C 1s XPS peak broadening after four cycles of CO intercalation/desorption

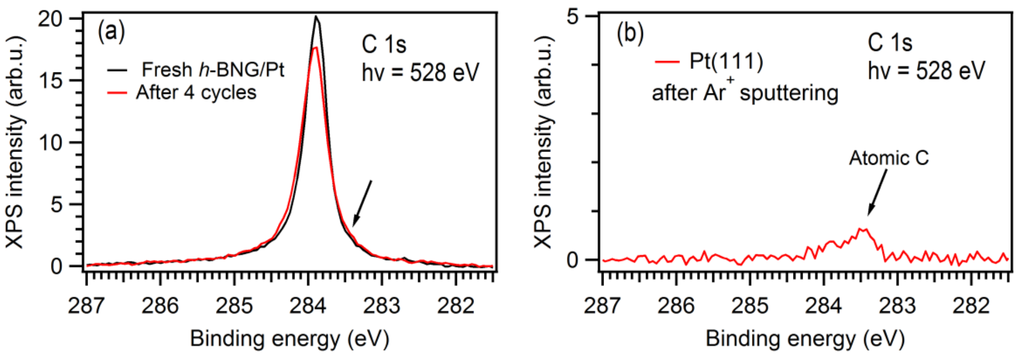

Fig. S6. (a) XPS C 1s spectrum for *h*-BNG 2D-layer on Pt(111) after the preparation and after 4 cycles of CO intercalation and desorption at T = 610 K. Peak broadening is clearly visible. The high BE side broadening can be ascribed to an increased C–Pt hybridization.[3] The low BE broadening (marked by arrow) can be attributed to the presence of atomic carbon left on the surface after CO dissociation. Such carbon peak is observed during Pt(111) cleaning procedure (after Ar^+ sputtering), as shown in panel (b).

1 R. Miranda, K. Wandelt, D. Rieger and R. D. Schnell, Angle-resolved photoemission of CO chemisorption on Pd(111), *Surf. Sci.*, 1984, **139**, 430–442.

2 M. Kinne, T. Fuhrmann, C. M. Whelan, J. F. Zhu, J. Pantförder, M. Probst, G. Held, R. Denecke and H. P. Steinrück, Kinetic parameters of CO adsorbed on Pt(111) studied by in situ high resolution x-ray photoelectron spectroscopy, *J. Chem. Phys.*, 2002, **117**, 10852–10859.

3 S. Rajasekaran, S. Kaya, F. Abild-Pedersen, T. Anniyev, F. Yang, D. Stacchiola, H. Ogasawara and A. Nilsson, Reversible graphene-metal contact through hydrogenation, *Phys. Rev. B*, 2012, **86**, 075417.
